# Social and individual factors associated with eating disorders risk among adolescents in secondary schools of Sicily (south-Italy)

**DOI:** 10.1186/s13034-025-00940-2

**Published:** 2025-07-14

**Authors:** Silvia Ruggieri, Rosalba Contentezza, Melania Casella, Achille Cernigliaro, Ilaria Cosentini, Gaspare Drago, Gianluca Lo Coco, Maria Rita Semola, Salvatore Gullo

**Affiliations:** 1 Institute for Biomedical Research and Innovation, National Research Council of Italy, Palermo, 90146 Italy; 2 Sicilian Order of Psychologists, Palermo, Italy; 3 Integrated Operative Unit of Clinical Pathology, Department of Services and Radiological Sciences, Sant’Antonio Abate Hospital, Trapani Provincial Health Authority, Erice, TP Italy; 4 Department of Health Services and Epidemiological Observatory (DASOE), Regional Health Authority, Palermo, Sicilian Region Italy; 5 Department of Psychology, Educational Science and Human Movement, University of Palermo, Palermo, Italy

**Keywords:** Eating disorders, Risk factors, EDE-Q, Adolescent, Gender role, Body image, Bullying, Epidemiological studies

## Abstract

Eating disorders (EDs) are complex medical conditions that primarily affect adolescents. In Italy, EDs are becoming a serious health concern, but the lack of up-to-date epidemiological data makes it difficult to assess their real impact across the country. Early identification of risk factors is crucial for timely intervention. A multicenter cross-sectional survey was conducted among 1,740 students aged 14–16 years in Sicily. Data on socio-demographic factors, EDs behaviors, social media usage, body appreciation, and bullying were collected through an electronic questionnaire. Regression models were used to test the role of gender and interaction effects with key risk factors. Based on Eating Disorder Examination-Questionnaire (EDE-Q) scores, 72.8% of participants were classified as “not at risk”, 17.9% as “at risk” and 9.3% as “at higher risk” of developing EDs using gender-related cut-offs. Girls were overrepresented in the at-risk and higher-risk groups (*p* < 0.01). Analysis revealed that lower Body Appreciation Scale (BAS) scores, higher BMI, and body comparison on social media predicted higher EDE-Q scores (*p* < 0.01). Gender interacted with several risk factors, including body appreciation (ß = 0.30. *p* < 0.01), alcohol use (ß = − 0.14, *p* < 0.01), and the perceived impact of the COVID-19 pandemic (ß = − 0.06, *p* < 0.05), with females showing stronger associations. This study highlights the increasing concern about EDs among Italian adolescents and emphasizes the need for targeted screening and early intervention. Understanding the interplay between gender, body image, and social behaviors is key to preventing EDs mitigating their long-term consequences.

**Supplementary Information:**

The online version contains supplementary material available at 10.1186/s13034-025-00940-2.

## Background

Eating disorders (EDs) are complex and serious medical conditions associated with high rates of morbidity and mortality that primarily affect pre-teen adolescents, adolescents and young adults [1, 2]. According to DSM-5, EDs are “characterized by a persistent disturbance of eating or eating-related behavior that results in the altered consumption or absorption of food and that significantly impairs physical health or psychosocial functioning” [3].

Eating disorders are a growing concern worldwide and represent an emergency in terms of public health due to the high rate of chronicity, hospitalization and medical comorbidity. The complexity of diagnosis and the several fragmented data collection make it difficult to assess EDs’ global prevalence, but it can be stated that EDs present a high and increasing prevalence worldwide [4, 5].

The Italian data, published during the pre-COVID-19 pandemic period, estimated that about 3.3 million subjects among general population suffer from an ED with a 0.2-0.8% prevalence for Anorexia Nervosa and 1-5% for Bulimia Nervosa [6]. However, this can be considered an underestimated value as many cases do not present to health services or do not have an appropriate diagnosis [7]. Furthermore, the health policies to contain the spread of SARS-Cov-2 during the pandemic have further impacted on vulnerable individuals, increasing the population suffering of EDs [8, 9].

The etiology of EDs is heterogeneous including biological, psychological, developmental, and socio-cultural factors [10, 11]. There is a considerable amount of research supporting the role of several risk factors in the onset and in the development of EDs. However, a recent systematic review [12] highlighted that most of the studies frequently evaluated only psychological risk factors associated with eating disorders onset; only a few studies analyzed the relationship between family factors or socio-cultural factors, such as alcohol use or the influence of social media, and eating disorders or protective factors such as sporting activities.

Biological factors include gender and age: girls and women are much more likely than boys and men to have EDs [13]. Although EDs are historically considered a female medical condition, recent studies have determined that EDs among males are not uncommon and are equally severe in symptom presentation [14]. Traumatic events are often considered to trigger EDs [15]. Studies of families have found that having a first-degree relative (like a parent or sibling) with an eating disorder increases a person’s risk of developing an eating disorder [16]. There are also psychological factors possibly causing the onset of EDs such as obsessive-compulsiveness personality traits [17]. Socio-cultural factors can also contribute: the increased society pressure through social networks plays a crucial role in the onset of EDs causing body-image dissatisfaction and internalization of “thin ideal” [18, 19]. The COVID-19 pandemic has had a considerable impact on the rates of probable eating disorders, as well as worsening of symptoms and general mental health. There is substantial evidence that vulnerable individuals were at greater risk of developing and progressing to eating disorders during the COVID-19 pandemic [8, 20, 21]. Studies on non-clinical samples (many of them with adolescent) have found that a significant percentage of people (20–30%) reported worsening of specific eating disorder symptoms (including binge eating, eating restriction, vigorous exercise) due to the pandemic. Moreover, higher percentages of people (30–60%) reporting concerns about eating, shape, and weight [22, 23].

In addition, the peculiar characteristics of adolescence and the pubertal development process are also critical factors in determining the age of onset, diagnosis, treatment and outcome of EDs in this age group [24, 25]. It should be noted that school environment may significantly impact on adolescents’ mental health, including the onset and progression of EDs [26, 27]. The interactions and experiences within these settings play a crucial role in shaping their mental well-being. Experiences of bullying phenomena within the school environment can further elevate the risk of developing EDs [28–30]. Thus, a correct knowledge of the relationships of the determinants in EDs offers the opportunity for early identification of high-risk groups.

The increase of the number of adolescents with EDs highlights the need to promote research on the underlying causes, to identify related risk factors which may guide the development of targeted, evidence-based prevention, early intervention, and treatment programs.

Thus, the primary aim of this study was to examine the role of individual, familiar and social factors on the risk of EDs onset in a population of adolescents aged 14–16 years resident in Sicily, a Mediterranean area of southern Italy.

Specifically, the study hypothesizes a positive association between individual factors (such as BMI, appreciation of one’s body, appearance comparison through social media, use of alcohol, smoking, self-harm episodes and physical exercise), familiar and social factors (as the presence of EDs in family, the impact of COVID and the involvement in bullyism and/or victimization episodes) with EDs risk behaviors as measured by the EDE-Q global score. Furthermore, the study hypothesizes that gender may significantly interact with the above-mentioned factors in predicting EDs risk behaviors.

## Materials and methods

### Sample recruitment

During the school year 2022/23, 15 secondary second degree schools of Sicily were involved on the basis of their Principals’ availability. The total population of the schools was 3,150 students. Among these schools, 69 classes were randomly selected for the study. 1,740 students received parents’ consent to participate and were involved to collect data about the risk factor for EDs onset (55.2% of total school population). Inclusion criteria for study participation were: age comprised between 14 and 16 years old; attending the first, second or third class.

### Ethical considerations

The study was approved by the CNR Research Ethics and Integrity Committee (May 22, 2024, n 0171964/2024) and by the Ethics Committee of the University of Palermo (May 30. 2024, n. 218/2024). Since the study involved minors, informed consent was obtained from both the parents of all subjects involved in the study. The study has been conducted following the Declaration of Helsinki principles. All the adopted procedures comply with the General Data Protection Regulation (UE 2016/679) and Italian laws concerning data protection. The respect of individual privacy concerning clinical data was granted.

### Questionnaire

The study was carried out through the submission of an electronic questionnaire in the presence of at least one researcher from the research team. The questionnaire was accessible online scanning a QR code. Students usually completed the questionnaire in about 40 min. The questionnaire consisted of an introductive section on demographic and socioeconomic data (gender, age, nationality, school institution and class attended, family educational level) followed by items investigating personal factors such as BMI, appreciation of one’s body, dysfunctional behaviors (self-harm, use of drugs, alcohol, etc.), family factors (level of education and presence of nutritional problems in parents) and social factors such as the influence of social and mass media, the comparison of one’s body image with that proposed by social media.

In order to investigate the presence of eating problems and related risk factors, the following questionnaires were administered:

The Eating Disorder Examination-Questionnaire (EDE-Q), a well-established self-report questionnaire to assess psychopathology and behaviors of EDs [31, 32]. In this study it has been used the validated full Italian version which has very good psychometric properties in terms of internal consistency and test-retest reliability, and good criterion validity [33]. EDE-Q is composed of 28 items scored using a 7-point, forced choice rating scale (0–6). Items are categorized into 4 subscales, with higher scores reflecting greater severity or frequency of core psychopathological features of ED: restraint, eating concern, weight concern, and shape concern. A global score is computed for each subject as the mean score of the four above mentioned subscales. To classify participants according to their risk of developing an ED, a gender-specific thresholds for distinguishing the “not at risk” to “at risk” category was applied. Specifically, a cut-off of 2.3 for females and 1.68 for males were used. The 2.3 threshold has been widely reported in literature to differentiate between individuals with and without ED risks in female samples [34], even though it has not been fully validated in clinical population [35]. For males, however, several studies have shown that the 2.3 threshold may underestimate ED risk. Consequently, we adopted the lower cut-off of 1.68, which has been proposed as more appropriate for identifying ED risk in male populations [36–38].

Furthermore, we selected an additional cutoff of 3.4 for both males and females to identify the most severe cases, consistently to previous evidences that found a mean EDE-Q score of 3.4 in a group of 264 Italian patients with an ED diagnosis [33]. In this study, “not at risk” group includes all individuals with a global EDE-Q score of less than 2.3 for females and 1.68 for males; girls in the “at risk” group have an EDE-Q score between 2.3 and 3.4 while the boys range between 1.68 and 3.4 for males. The remaining subjects are included in the “at higher risk” group.

The Body Appreciation Scale 2 (BAS-2) [39, 40], a psychometric tool designed to measure individuals’ positive attitudes towards their own bodies. It assesses the extent to which individuals accept, respect, and hold favorable opinions about their bodies, while also rejecting societal ideals of stereotypical beauty. The BAS-2 questionnaire consists of 10 items rated on a Likert scale (1 = Never; 5 = Always), where respondents indicate their level of agreement with statements related to body appreciation. The final score is computed summing the items. Higher scores indicate a more positive body image perception. In this study, we used the validated full Italian version of BAS-2 which has been shown to have good consistency and adequate construct validity [41].

The Florence Bullying/Victimization Scale (FBVS) [42], a comprehensive assessment tool designed to evaluate the prevalence and nature of bullying and victimization behaviors among individuals, typically in educational settings. FBVS has been shown to have a robust factor structure and good reliability [42]. Respondents are asked to indicate how often they have engaged in or been subjected to various bullying behaviors over a specific period using a 5-point scale (1 = Never; 5 = Several times a week). A final score, one for perpetration and one for victimization during the previous 2–3 months, are computed by means of three subscales: physical behaviors, verbal behaviors and indirect-relational behaviors. In this study, we choose at least one experience of perpetrated and/or suffered bullying as a cutoff in order to categorize the students into bullies, victims, involved in both bullying others and being bullied (bully-victims) and not involved.

### Statistical analysis

Sociodemographic variables were collected through questions with binary (“yes” or “no”) responses. Categorical variables are reported as numbers and percentages for summary statistics. Normally distributed continuous variables were reported as mean ± SD, while non-normally distributed variables are shown as median and interquartile range (IQR). Kolmogorov-Smirnov was performed to test the normality of continuous variable distributions. Kruskal-Wallis test was used to study the differences in non-normal variables, whereas t-test and ANOVA were used to test between group differences for normally distributed variables. A chi-squared test or Fisher’s exact test, when appropriate, were used to identify differences in categorical variables. Generalized linear model (GLM) was used to examine the contribution of gender, personal, familiar and social risk factors to EDs and the effects of interaction between these factors and gender. Specifically, the adopted model consists of three steps: in the first one gender was entered as dichotomous predictor (female = 0 male = 1) and EDE-Q scores as continuous outcome. In the second step the following factors were added as continuous predictors: BMI, BAS scores, body comparison throughout social media; while use of alcohol, smoke, presence of self-harm episodes, presence of physical exercise, presence of ED in family, impact of COVID, presence of bullyism, and victimization were added as dichotomous variables. Sensitivity analyses stratifying for gender and for bullism/victimism were conducted. The results are presented using a Forest plot generated with the R package “ggplot2”. In addition, in step three the interactions between gender and the previously reported factors were added. The results are presented with a two-way interaction plot to evaluate interactions between the effects of gender and significant risk factors on EDE-Q scores. All tests were conducted at a nominal alpha error of 0.05. All the analyses were performed in SPSS v.22 or R v. 4.3.1.

## Results

### Characteristics of risk and high-risk groups in the overall sample

The study included 1,740 adolescents (796 girls, 928 boys and 16 non-binary defined) recruited from the public secondary, second degree, schools in Sicily. The sample includes adolescents having median age 15.0[IQR: 14.0;15.0] and most of them were of European origin (*N* = 1,688, 97.0%). On the basis of different gender-related cut-off and to account for these differences, we initially focused on the male subgroup, applying the 1.68 threshold to classify participants as “not at risk” or “at risk”. Among those categorized as “at risk”, the median score was 2.38 [1.96; 2.93]. Notably, using the traditional cut-off of 2.3 would have misclassified 24.7% (*n* = 77) of these individuals as “not at risk”. Based on these findings, we adopted sex-specific threshold to better asses ED risk. Consequently, partecipants were categorized into three groups according to their EDE-Q global score: “not at risk” (EDE-Q < 2.3 for females; EDE-Q < 1.68 for males), “at risk” (2.3 ≤ EDE-Q ≤ 3.4 for females; 1.68 ≤ EDE-Q ≤ 3.4 for males) and “at higher risk” (EDE-Q > 3.4 for both males and females) (Table 1).

The majority of participants (*N* = 1267, 72.82%) were classified in the “not at risk” group (EDE-Q score = 0.48[0.15;1.06]), 17.93% (*N* = 312) were “at risk” (EDE-Q score = 2.60[2.30;2.94]), and the remaining 9.3% (*N* = 161) had an EDE-Q global score over 3.4 (EDE-Q score = 3.96[3.71;4.33]) and were included in the “at higher risk” group.

**Table 1:** Demographic and clinical characteristics of the three groups: “not at risk” (females EDE-Q < 2.3– males EDE-Q < 1.68), “at risk” (2.3 ≤ EDE-Q ≤ 3.4 for females and 1.68 ≤ EDE-Q ≤ 3.4 for males) and “at higher risk” (EDE-Q > 3.4). Overall p-values across the groups are provided, along with pairwise comparisons relative to the reference group (“not at risk”-naR)

	not at RISK	at RISK	at HIGHER RISK	STATISTIC COMPARISON WITH naR GROUP
	*N* = 1,40 (100%)			
N(%)	1267 (72.82%)	312 (17.93%)	161 (9.25%)	
Age, Median[IQR]	15.0 [14.0;15.0]	15.0 [14.0;15.0]	15.0 [14.0;15.0]	
Italian Nationality, N(%)	1245 (98.3%)	311 (99.7%)	158 (98.1%)	
Gender– N(%):				OV^***^, X^2^ = 122.5; aR^***^, X^2^ = 20.59; aHR^***^, X^2^ = 114.3
Females	497 (39.2%)	165 (52.9%)	134 (83.2%)	
Males	760 (60.0%)	143 (45.8%)	25 (15.5%)	
Others	10 (0.79%)	4 (1.28%)	2 (1.24%)	
^1^EDE-Q subscales, Median[IQR]:				
1.Restraint	0.20 [0.00;1.00]	2.80 [1.80;3.60]	4.40 [3.80;5.00]	OV^***^, H = 1000.3; aR^***^, H = 809.08; aHR^***^, H = 1202.4
2. Eating concerns	0.20 [0.00;0.60]	2.00 [1.40;2.60]	3.60 [3.00;4.20]	OV^***^, H = 911.83; aR^***^, H = 702.73; aHR^***^, H = 1200
3. Shape concern	0.71 [0.14;1.57]	3.43 [2.86;3.71]	4.57 [4.14;4.71]	OV^***^, H = 1122; aR^***^, H = 927.44; aHR^***^, H = 1294
4. Weight concern	0.40 [0.00;1.00]	2.50 [2.00;2.80]	3.80 [3.40;4.20]	OV^***^, H = 1089.8; aR^***^, H = 894.26; aHR^***^, H = 1324
^2^BMI, Median[IQR]	20.0 [18.5;22.1]	23.4 [20.6;26.0]	23.1 [20.8;25.4]	OV^***^, H = 1007; aR^***^, H = 945.81; aHR^***^, H = 855.28
^3^BAS Total score, Median[IQR]	39.0 [32.0;45.0]	25.0 [20.0;32.0]	19.0 [14.0;26.0]	OV^***^, H = 557.81; aR^***^, H = 405.16; aHR^***^, H = 584.76

^1^EDE-Q: Eating disorders examinations questionnaire

^2^ BMI: Body Mass Index (Kg/cm^2^)

^3^ BAS: Body Appreciation Scale

H: Kruskal-Wallis test statistic; X^2^: Chi-squared test statistic; OV: Overall; naR: “not at risk”; aR: “at risk” vs. “not at risk” comparison; aHR “at higher risk” vs “not at risk” comparison

*p < 0.05; **p < 0.01; ***p < 0.001; no statistically significant comparisons are omitted

### The role of gender, BMI and body appreciation

The gender distribution was different (*p* < 0.01): the “not a risk” group included a higher percentage of boys (60.0%), while the other two groups showed a higher percentage of girls (59.2% and 83.2% in the “at risk” and “at higher risk” groups, respectively).

The three groups showed significant differences in all the four EDE-Q scales. Adolescents belonging to “at higher risk” group had higher scores on concern about body shape and weight and internalization of the thinness ideal than peers in “at risk” and “not at risk” groups (*p* < 0.01); moreover students “at risk” had higher scores in all the four scale than student in “not at risk” group (*p* < 0.01).

Mean BMI was 21.4 kg/m2 (± 3.74 SD) in the entire sample, and it was higher in both “at risk” and “at higher risk” groups compared to the “not at risk” group (*p* < 0.01).

According to the WHO standards [43] (underweight < 18.5 kg/m2, normal 18.50–24.99 kg/m2, overweight ≥ 25 kg/m2, obese ≥ 30 kg/m2), the students were subdivided into underweight (UW, 20.0%), normal weight (NW, 65%), overweight (OW, 12.1%) and obese (OB, 2.9%). Specifically, 76.5% of obese adolescents and 53.1% of overweight adolescents had an EDE-Q score above 2.3 (merging at “risk” and at “higher risk” groups), whereas 74.4% of those with BMI in the normal range had a “not a risk” EDE-Q score. Surprisingly in the latter group also the 90.5% of underweight adolescents fell.

Finally, body appreciation was found to be associated with the different levels of ED risk, subjects belonging to “at higher risk” group showed a significant lower BAS score compared to the “not at risk” and the “at risk” groups (p_s_<0.01), this latter group also showing lower body appreciation than individuals with EDE-Q scores in “not at risk” range (*p* < 0.01).

### The role of personal, familiar and social factors

Several other personal factors were associated with EDE-Q scores (Table 2).

**Table 2:** Overview of personal, familiar and social factors across three groups: “not at risk” (naR: EDE-Q < 2.3 for females and EDE-Q < 1.68 for males), “at risk” (aR: 2.3/1.68 ≤ EDE-Q ≤ 3.4) and “at higher risk” (aHR: EDE-Q > 3.4). Overall p-values across the groups are provided, along with pairwise comparisons relative to the reference NaR group

	not at RISK	at RISK	at HIGHER RISK	STATISTIC COMPARISON WITH naR GROUP
	*N* = 1,740 (100%)			
N(%)	1267 (72.82%)	312 (17.93%)	161 (9.25%)	
Self-harm [Yes], N(%)	124 (9.79%)	57 (18.3%)	76 (47.2%)	OV^***^, X^2^ = 162.57; aR^***^, X^2^ = 16.92; aHR^***^, X^2^ = 162.97
Alcohol consumption [Yes], N(%)	619 (48.9%)	202 (64.7%)	105 (65.2%)	OV^***^, X^2^ = 35.64; aR^***^, X^2^ = 24.68; aHR^***^, X^2^ = 14.65
Smoking [Yes], N(%)	288 (22.7%)	128 (41%)	71 (44.1%)	OV^***^, X^2^ = 64.42; aR^***^, X^2^ = 42.24; aHR^***^, X^2^ = 33.53
Drugs consumption [Yes], N(%)	74 (5.84)	35 (11.2)	20 (12.4%)	OV^***^, X^2^ = 17.03; aR^**^, X^2^ = 10.44; aHR^**^, X^2^ = 9.02
Sport Activity (1–2 times/week) [Yes], N(%)	808 (63.8%)	185 (59.3%)	91 (56.5%)	
Familiar educational level, N(%):				aR^*^, X^2^ = 8.95
High	252 (20.8%)	57 (19%)	28 (18.1%)	
Medium	556 (46.0%)	116 (38.7%)	70 (45.1%)	
Low	402 (33.2%)	127 (42.3%)	57 (36.8%)	
Familiar with obesity, N(%)	275 (21.7%)	117 (37.5%)	66 (41.0%)	OV^***^, X^2^ = 51.9; aR^***^, X^2^ = 32.63; aHR^***^, X^2^ = 28.18
Familiar with EDs, N(%)	145 (11.4%)	56 (17.9%)	47 (29.2%)	OV^***^, X^2^ = 1.07; aR^**^, X^2^ = 8.96; aHR^***^, X^2^ = 37.16
^1^FBVS, N(%):				OV^***^, X^2^ = 62.37; aR^***^, X^2^ = 17.01; aHR^***^, X^2^ = 51.46
BULLIES	194 (15.3%)	57 (18.3%)	12 (7.45%)	
UNINVOLVED	876 (69.1%)	180 (57.7%)	91 (56.5%)	
VICTIMS	142 (11.2%)	52 (16.7%)	50 (31.1%)	
VICTIMS/BULLIES	55 (4.34%)	23 (7.37%)	8 (4.97%)	
Social network usage [Yes], N(%)	1259 (99.4%)	310 (99.4%)	161 (100%)	
Time on social, N(%):				OV^***^, X^2^ = 41.16; aR^***^, X^2^ = 16.98; aHR^***^, X^2^ = 29.04
More than 7 h	117 (9.42%)	42 (13.8%)	36 (23.1%)	
from 4 to 6 h	370 (29.8%)	105 (34.5%)	48 (30.8%)	
from 2 to 3 h	560 (45.1%)	133 (43.8%)	56 (35.9%)	
Less than 1 h	195 (15.7%)	24 (7.89%)	16 (10.3%)	
Instagram, N(%)	1188 (88.2%)	283 (90.7%)	140 (87.0%)	
TikTok, N(%)	1054 (83.2%)	280 (89.7%)	149 (92.5%)	OV***, X^2^ = 16.09; aR**, X^2^ = 7.71; aHR**, X^2^ = 8.73
Comparison with social [Yes], N(%)	323 (26.9%)	154 (53.8%)	111 (74.0%)	OV***, X^2^ = 176.74; aR***, X^2^ = 75.61; aHR***, X^2^ = 133.36
COVID-19 impact [Yes], N(%)	395 (31.2%)	209 (67.0%)	132 (82.0%)	OV***, X^2^ = 246.05; aR***, X^2^ = 134.42; aHR***, X^2^ = 156.31

^1^ FBVS: Florence Bullying-Victimization Scale

X^2^: Chi-squared test statistic; OV: Overall; naR: “not at risk”; aR: “at risk” vs “not at risk” comparison; aHR “at higher risk” vs “not at risk” comparison

*p < 0.05; **p < 0.01; ***p < 0.001; no statistically significant comparisons are omitted

The 18% of participants “at risk” and 47% of those “at higher risk” declared episodes of self-harm in the last 12 months (against the 10% of subjects “not at risk”, *p* < 0.01). Subjects in the two former groups were also more likely to consume alcohol (65% in both at risk groups vs. 49% in “not at risk” group; *p* < 0.01) and to smoke (44% and 41% respectively vs. 23% in “not at risk” group; *p* < 0.01). Students “at risk” and “at higher risk” also reported substance use (11% and 12% respectively) in a significantly higher percentage than their peers in “not at risk” group (6%; *p* < 0.01). Although engaging in sport activity was frequent throughout the entire sample, the subgroup having a “not at risk” EDE-Q score was more likely to do physical activity at least once a week (64%) compared to “at risk” (59%) and at “high risk” (57%) groups ( *p *no statistically significant). In order to familiar factors, educational level of the parents was considered as the family educational level, reporting the highest level of education between mother and father. The educational level was different among the three groups: the lowest educational level (42.3%) was found in parents of students “at risk” (*p* < 0.05). As expected, family history of eating problems had an effect on outcome, subjects “at higher risk” to develop an ED are more likely to have a parent with obesity (41%) and/or with EDs (29.2%) in comparison with “at risk” and “not at risk” subjects (*p* < 0.01).

Social factors also play a significant role. As shown in Tables 2 and 31% of adolescents belonging to the “at higher risk” group are mainly involved in suffering bullying (31%), a percentage significantly higher than those shown by “at risk” and “not at risk” group (17% and 11% respectively, *p* < 0.01). On the contrary, adolescents belonging to the “at risk” and “not at risk” groups are mainly involved in bullying others (18% and 15% respectively, *p* < 0.01). Most of the subjects in the “normal” group are not involved in acts of bullying (69%).

More than 99% of students use the social networks. Time spent on social networks was different among the subgroups (*p* < 0.01), the 23.1% of adolescents in the “at higher risk” group spending more than 7 h on social media. Only few students (13.5% of the total sample) use social media less than one hour, while most of them use social media at least more than 2 h. Instagram and TikTok are the most used social media. In particular, TikTok is highly used among adolescents with an EDE-Q score over the 2.3 cut-off (*p* < 0.01). In addition, the percentage of adolescents comparing their body with others in social media is higher (*p* < 0.01) in “at risk” (53.8%) and “at higher risk” (74%) groups compared to “not at risk” group (28%).

Finally, subjects in the “at risk” and “at higher risk” groups, 67% and 82% respectively, perceived a direct influence from the COVID-19 pandemic on their relationship with one’s own body and on nutrition, a percentage clearly higher than that reported by the subjects in the “not at risk” group (31%; *p* < 0.01).

### Interaction between gender and factors associated with ED risk

The preliminary analyses suggested that gender plays a noticeable effect on outcome. We also explored the effects of risk factors on EDE-Q scores and the effects of interaction between risk factors and gender by applying a linear regression model. In the following results, gender was entered as predictor in the first step, other risk factors were added in the second step, and the interaction in the third one. Regression models presented separately for each gender are detailed in Supplementary Materials (Table S1).

**Fig. 1:**
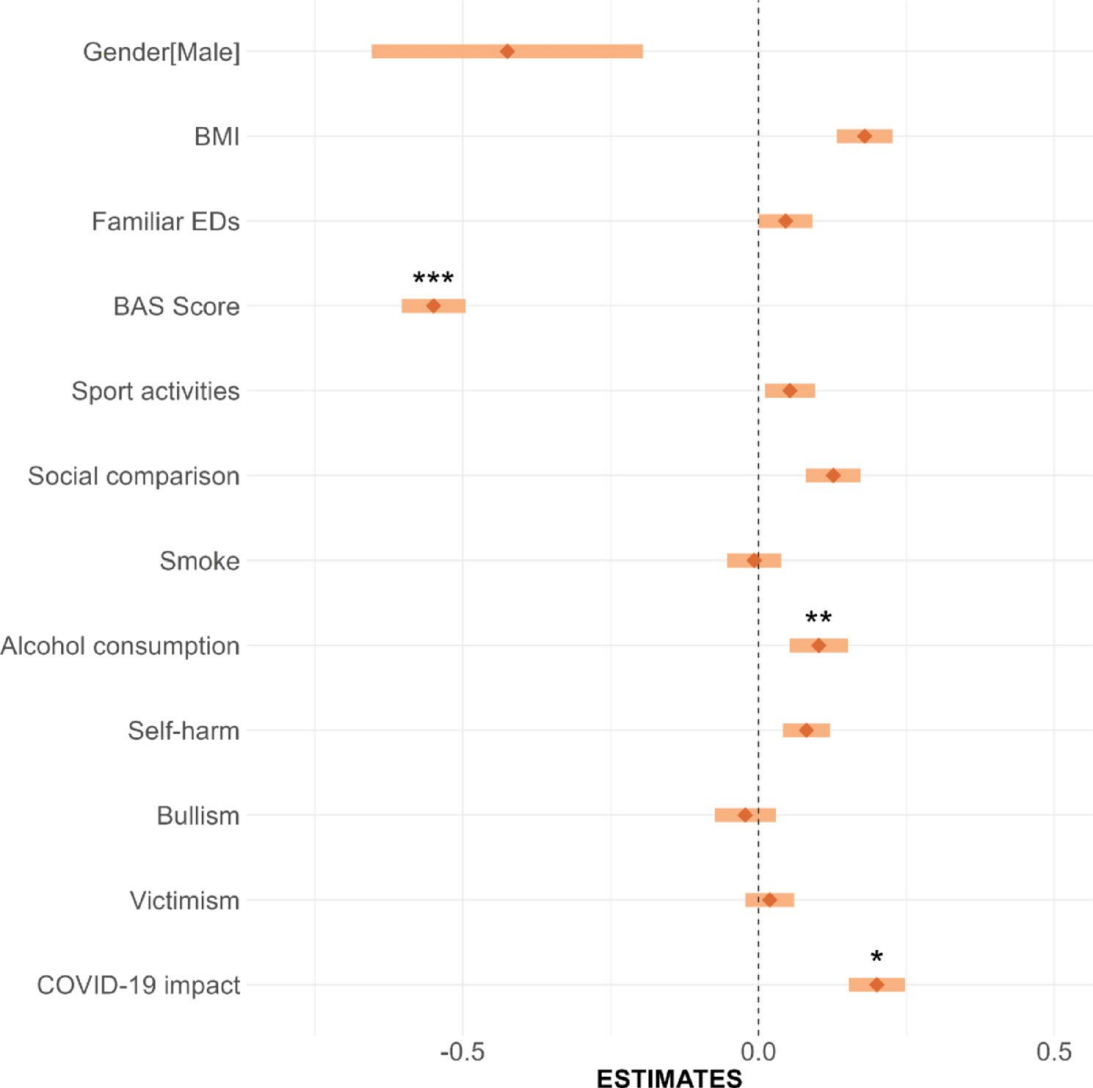
Forest Plot illustrating the Standardized Beta Coefficients for Personal, Familial, and Social Factors in Relation to Eating Disorder Risk (EDE-Q score). Factors include Gender (Male), BMI, Familial Eating Disorders (EDs), BAS Score, Sport Activities, Social Comparison, Smoking, Alcohol Consumption, Self-harm, Bullying, Victimization, and the Impact of COVID-19. Significance levels of the interaction terms are indicated as follows: *p < 0.05, **p < 0.01, ***p < 0.001

Over and above the effect of gender (ß = − 0.4; *p* < 0.01, reference females), the addition of risk factors in the second step, as well as the addiction of interactions in the third step, resulted in a significant improvement of explained variance (from 17 to 63% and to 65%, respectively). Figure 1 presents the results from the regression model, incorporating all risk factors and assessing whether the interaction term with gender is significant.

In the second step low BAS scores (ß = − 0.55, *p* < 0.01), high BMI (ß = 0.18, *p* < 0.01), and more frequent behaviors such as body comparison throughout social media (ß = 0.13, *p* < 0.01), use of alcohol (ß = 0.10. *p* < 0.01), self-harm episodes (ß = 0.09, *p* < 0.01) and physical activity (ß = 0.06, *p* < 0.05), significantly predicted higher EDE-Q scores, confirming their potential role as risk factors. Moreover, the presence of ED in family (ß = 0.05, *p* < 0.05) and the perceived impact of COVID pandemic (ß = 0.20. *p* < 0.01) had an effect on the increase of EDE-Q scores. Results from the third step showed that gender presented significant interactions with BAS scores (ß = 0.30. *p* < 0.01), use of alcohol (ß = − 0.14, *p* < 0.01) and impact of COVID-19 (ß = − 0.06, *p* < 0.05). The other interactions did not reach statistical significance despite they showed an interesting trend towards significance: gender x smoke (ß = 0.05, *p* = 0.06), gender x victimization (ß = 0.04, *p* = 0.08), and gender x body comparison (ß = 0.04, *p* < 0.08). Finally, gender did not show any significant interaction effect for BMI, physical exercise, bullyism and presence of ED in family. No specific risk factors were identified as uniquely associated with either being a perpetrator or a victim of bullying, suggesting that similar psychosocial and behavioral variables may contribute to EDs across both groups (results are shown in Figure S1).

**Fig. 2:**
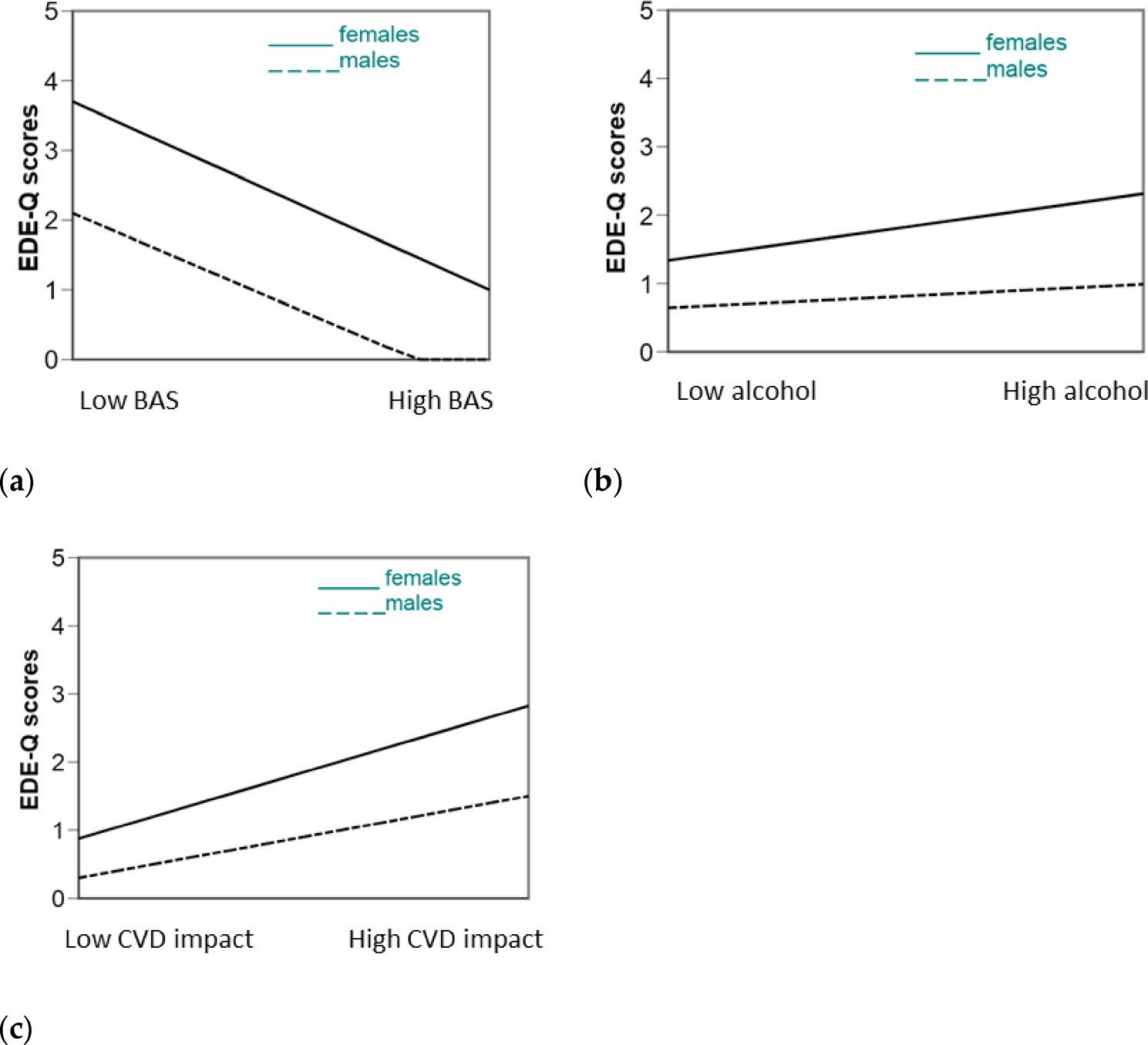
Interaction analysis results. **a** Interaction gender x BAS scores; **b** Interaction gender x alcohol use; **c** Interaction gender x COVID-19 impact

Figure 2A and C showed the results of two-way interactions: interpretation of plots suggests that the effect of BAS scores on EDE-Q scores is more pronounced for female, low score in BA corresponding to high risk of ED, while the flatter line for males indicates a less pronounced effect of BAS on EDE-Q score (2 A). Similarly, among females the risk of EDs was more influenced by alcohol use and perceived impact of COVID-19 on one’s body and nutrition with respect to male peers (Fig. 2B and C).

The BMI showed a significant positive correlation to EDE-Q score in both male and female students respectively (ß = 0.48 and ß = 0.38; p_s_ < 0.01). Higher BMI was correlated to higher EDE-Q scores and the effect of BMI was higher in males than in females (ß = 0.10. t = − 2.06, *p* < 0.05). Conversely, negative associations were found for BAS scores and EDE-Q scores: in both groups the association was significant for males and females respectively (ß = − 0.59, ß = − 0.70; p_s_ < 0.01); low body appreciation predicted higher EDE-Q scores, and this association was stronger in females than in males (ß = 0.11, t = − 2.27, *p* < 0.05). Sensitivity analyses for those variables that had interactions with gender approaching significance showed that the effect of smoking was larger in males than in females (ß = 0.07 and ß = 0.01, respectively); the effect of body comparison was significant for both sexes but larger in females than in males (ß = 0.12 and ß = 0.08, respectively). Similarly, the effect of being a victim was also larger in females than in males (ß = 0.09 and ß = 0.01, respectively).

## Discussion

This cross-sectional survey explored the role of several risk factors that may increase the likelihood that a person might develop an eating disorder in a sample of 1,740 middle adolescents attending secondary schools in the southern Italy. Several reasons led us to investigate eating attitudes among adolescents of this age range.

The main result of our sample of non-clinical population of adolescents is that a large portion of our sample reports a risk of EDs (27.2%). Among these 34% reported an EDE-Q score similar to clinical population with a EDs diagnosis (9% of the total sample). Our findings are similar to those found in other countries. A recent meta-analysis that considers a population of more than 63,000 subjects, collecting 32 studies from 16 countries, showed that the overall proportion of children and adolescents with disordered eating was about 22% (95% CI, 18.84-26.09%). Similarly, a study published in 2024, in which EDE-Q 6.0 was administered, reported a similar rate of 21.5% among undergraduate Pakistan students (18–24 year-old) [44].

It should be noted that a risk of EDs does not always indicate the presence of an ED and that not all adolescents who reported disordered eating behaviors will necessarily be diagnosed with an ED [45]. However, this data is alarming because the proportion of young people with disordered eating appears to increase with increasing age and empirical evidences support the hypothesis that difficulties in eating behaviors in adolescence may predict outcomes associated with EDs in early adulthood [45].

Consistent with previous research and meta-analyses [13, 46], EDE-Q scores were higher among females when compared to males, showing a higher prevalence of girls in “at risk” and “at higher risk” subgroups. From an epidemiological point of view, EDs in males, although showing a lower impact than in females, have been steadily increasing in recent years. Although they share some clinical aspects, ED in males differ from those found in females with respect to some concepts [47].The gender differences reported in the literature depends on the eating disorder behaviors under investigation: girls or women are more likely than boys or men to report weight dissatisfaction, dieting for weight control. Conversely, boys or men are more likely to report use of excessive exercise for weight control [13]. Beyond the well-known prevalence of EDs in females, in this study we also explored the indirect effect of gender on EDE-Q scores and found that gender plays a significant effect in the interaction with body appreciation, alcohol use, and impact of the pandemic, while it shows a less strong interaction (which in any case approaches significance) with smoking, being a victim of bullying, victimization and body comparison through social media. Even when considering a lower cutoff (EDEQ > 1.68) to define males as “at risk”, as suggested by recent literature.

As age and gender, also BMI is commonly described as to be a risk factor for the onset and maintenance of EDs [45, 48–50]. In this study, both “at risk” and “at higher risk” subgroups, boys and girls, are mainly characterized by overweight and obesity. Probably, OB and OW subjects internalize weight-based stigma originating from sources such as media and social networks and thus may have greater concerns about body image and a greater investment in weight loss [51]. On the contrary, underweight is associated with lower EDE-Q values as if, in a culture that praises weight loss and thinness, regardless of the path followed to reach the final goal, participants may not even understand their discomfort, considering be underweight as ‘normal’.

Among the investigated factors positively associated with EDE-Q, body dissatisfaction and relative self-esteem played a significant effect. Several studies highlighted the association between body dissatisfaction and the risk of developing disordered eating behaviors, particularly in adolescents and young adults [51–55]. A remarkable percentage of adolescents included in “at risk” and “at higher risk” subgroups reported significant body image concerns; and this, according to literature [56], was particularly pronounced among females, who showed greater dissatisfaction in relation to higher EDs. Our results support the role of body nonacceptance as a factor strongly associated with the risk of EDs and its prevalence in females. Moreover, our study also highlights how body image and EDs risks are correlated in males too (Table S1).

Among the other risk factors, parental eating behaviors play an important role as demonstrated in our model in which familiarity for obesity and EDs is associated with higher EDE-Q values. The family context and background have been extensively evaluated in literature as risk factor for the development of EDs [57, 58]. In fact, a strong association between parental weight status and risk of childhood obesity has been demonstrated [59]. Notably, parents with a history of obesity or EDs significantly impact their offspring’s risk of developing EDs from an early age [59–62]. While much of the research has focused on parental involvement, Steinhausen et al. demonstrated that having a sibling with an ED is also a significant predictor of developing EDs [57]. Our findings are in line with the existing literature, indicating that parental obesity and parents with EDs history increase the probability to develop EDs regardless of gender. In fact, in literature, in both cases, parental obesity and parents with EDs, are implicated in the etiology of EDs [63, 64]. Even if family environment is not the main risk factor for developing EDs, it could be considered among the most important ones. Therefore, we suggest investigating on parental dietary behaviors as a specific individual factor.

As concerns parental educational level (PEL), we found that higher value of family degree is positively associated with higher EDE-Q score; PEL is thought to influence children’s development and behavior in many ways [64]. Although many studies support an increased prevalence of EDs in families with higher PEL [65–69], conflicting results are present in literature. In fact, several studies showed no differences in lifetime ED prevalence based on PEL [70–72]. Such divergent results suggest that the relationship between PEL and EDs may be complex and depending on several factors.

Moreover, the rate of 23% of students with EDE-Q > 2.3 (females) or EDEQ > 1.68 (males) reported in our study is consistent with previous researches: in fact, since the post-pandemic period there has been an increase in the incidence of EDs and a lowering of the age of onset [73]. Community studies conducted before the pandemic in youths (2010-18) yielded prevalences of disordered eating behaviors ranging from 14 to 19% [74].

In order to characterize the several factors that could produce an increased risk for EDs, we also collected data on sport activities. Physical activity generally provides significant health and mental benefits [75]. Given that EDs are often accompanied by mental health comorbidities such as anxiety, depression, body image issues, and obsessive-compulsive disorders, physical activity could serve as a beneficial therapeutic support to ED treatment [76]. However, several studies have indicated that excessive exercise can become a symptom of EDs [77, 78]. Individuals with EDs often engage in extreme physical activity as a compensatory behavior to reduce food intake or prevent weight gain [79, 80]. This tendency is particularly prevalent in males, where compulsive exercise can become the primary method of weight control, overshadowing other symptoms [81]. However, the differing body ideals between females and males result in distinct behaviors as concerns physical activity. Women typically strive to achieve a certain body ideal, while men focus more on low body fat and muscularity [82, 83]. These findings are confirmed by our results, which did not identify physical activity as a significant variable for females but recognized it as a risk factor for males.

Sociocultural pressure to be thin and the widespread use of social media may increase body dissatisfaction and the desire to be thin, thus making adolescents more vulnerable to EDs [84, 85]. In line with this evidence, our results show how social media use and online social comparison are positively associated with EDs. However, our study design did not allow us to differentiate between specific types of social media engagements, such as passive scrolling, exposure to body image-focused content, or engagement with posts that subtly promote EDs attitudes. Nonetheless, some inferences can be drawn from the platforms most frequently used by partecipants. In our sample, TikTok and Instagram emerged as the most common platforms. While Instagram often reflects curated aspects of real-life interactions, TikTok’s algorithm is highly responsive to users behaviors showing contents catered to their own interests and self-representations [86]. Consequently, TikTok may intensify exposure to potentially harmful content without requiring active user interactions. This exposure could play a significant role in reinforcing body dissatisfaction and EDs, particularly among vulnerable adolescents [87].

Moreover, the amount of mobile screen time spent by our adolescents, spending over 2–3 h per day until 7 h per day, can be a cause of increasing levels of stress and anxiety as demonstrated by an experimental study conducted to investigate the effect of self-monitoring limited social media usage on psychological well-being. The results demonstrated that limiting social media usage to 30 min may significantly improve psychological well-being, decreasing depression and increasing positive feelings [88]. Even if it was suggested that an extensive use of mobile and social media can be a characteristic of problematic users, the link between social media usage and psychological characteristics of an adolescent has multiple causes and further research is needed [89].

Measures of alcohol consumption and smoke differed in males and females. According to literature [90], our results indicate that alcohol consumption is associated with higher EDE-Q values and to female gender. In fact, alcohol is particularly common in young women with EDs and can be used as an appetite suppressor and as a compensatory behavior to avoid food [91, 92]. With regard to smoke, our results show that smoke is more associated with male gender. As confirmed in literature, males are more likely to report smoking than females [93–95].

EDs are associated with various psychopathologies including self-harm which often occurs in subjects struggling with an ED [96]. The most common self-injury behaviors include cutting, scratching, burning, hair pulling or the consumption of harmful substances or objects [97]. Self-injury behaviors in the case of EDs include self-induced vomiting, laxative or diuretic misuse, excessive exercise, restricting behavior following a binge or eating until the point of discomfort or pain [98]. In our sample, self-injury is common among subjects included in the subgroup “at higher risk” reporting self-harm behaviors. Our findings are in line with the existing literature, showing a positive association between self-harm and EDs [99, 100].

To better characterize the several risk factors for the onset and development of EDs, we also investigated the association between bullying profiles (victims, bullies, and bully-victims) and the risk for EDs among adolescents. Bullying happens when “aggressive, intentional acts carried out by a group or an individual repeatedly and overtime against a victim who cannot easily defend him or herself” [101]. Bullying acts can be physical (i.e. hitting, pushing, kicking, etc.), verbal (i.e., name-calling, mocking, laughing), or relational (i.e., threatening, socially isolating a peer) [102]. It is a form of peer violence that is mainly prevalent in schools, in particular in secondary schools where bullying seems to be less perceived by students [103]. According to literature [104], in this study we categorized the students into: (i) bullies, i.e., subjects engaged in aggressive behaviors; (ii) victims, subjects who have become targets of such behaviors; (iii) bully-victims, perpetrating and experiencing violence simultaneously and (iv) not involved.

Our findings show that bullying victims, especially boys, are at a higher risk of developing EDs. Probably the victims are often bullied by peers because they are perceived as overweight or not fitting societal beauty standards [105]. This may be influenced by media and societal standards that promote a thin body ideal for girls and a muscular definition for boys [106]. Although bullying in all its forms appears to be more prevalent among males in our data, we did not identify distinct patterns of risk factors that clearly differentiate perpetrators from victims. Despite the ongoing challenge in obtaining unambiguous data on the prevalence and types of bullying, the continuous investigation of the prevalence and factors associated with the phenomenon is a necessary starting point for introducing interventions and preventive measures into Public Health programs [107].

Finally, the influence that the COVID-19 pandemic had on EDs deserves specific attention for several reasons. Several studies indicate an association between the COVID-19 pandemic and EDs’ development or exacerbation in youth, and a decrease in age of onset among school aged children and adolescents [20, 21]. In the present study, the answers to the question “Do you feel that the pandemic has affected your relationship with food and your perception of your body?” also suggest an increasing risk of developing of EDs with no gender limitation. These findings align with the international literature, which has documented an increased risk of eating disorders (ED) following the pandemic and lockdown [22], as well as an increase in the need for ED-related care during the pandemic [20, 108]. Previous Italian data supported similar evidences [8], showing that in Italy emergency room admissions and hospitalizations have doubled in the period 2021–2022, and in 2023 the Ministry of Health reported a 40% increase in new cases [73]. It has been hypothesized that prolonged social isolation and restraint in the home environment may represent both a stressogenic condition and a facilitating factor for binge eating and restrictive/compensative behaviors, and for EDs in general.

The results of this study highlight several key points. First, the high percentage of students at risk underlines the need to maintain a strong focus on this issue. Schools and other involved institutions should equip themselves with effective tools to detect this risk early on. Recommended strategies include implementing screening programs, establishing counseling centers, and providing information and training services. These measures, if consistently applied, can be useful in the early identification of individuals at risk, for whom preventive interventions would significantly improve the chances of recovery. Prevention efforts directed at students, their families, and teachers should focus on topics such as body appreciation, impact of social media on body comparison, ideals of thinness, and should also aim to develop skills to recognize other warning signs of eating disorders risks—such as low self-esteem, excessive physical activity, alcohol abuse, dysfunctional dieting, and unhealthy family eating habits.

### Strength and limitation

This study investigated the risk factors of EDs. A key strength is the opportunity to contribute to national data collection on EDs risks in adolescence, in the post-pandemic period, through a large and gender well-balanced sample.

Data about male with EDs are still scarce, this population is usually underrepresented and, in several studies, males are either excluded or combined with females. In this study, collecting data by means of EDE-Q, we are able to report estimates for gender showing that male gender is associated to specific risk factors, differently from female ones. Furthermore, the good balance between males and females allowed us to also explore gender differences on the investigated outcomes and the interaction with several other important aspects (personal, family and social factors) potentially related to risk of EDs.

The main results of the study are based on above mentioned gender related EDE-Q cut-offs through which we identified three distinct subgroups: “not a risk”, “at risk” and “at higher risk”, taking into account gender differences. Various studies have been conducted to define an appropriate cut-off for females, with findings suggesting a score of 2.3 [34, 109]. Moreover, Schaefer et al. [36] have investigated the accuracy of EDE-Q score in classifying male subjects with EDs, detecting a global EDE-Q score of 1.68 as the threshold for distinguishing between clinical and healthy individuals. Several studies identified a lower EDE-Q cut-off to effectively distinguish individuals with EDs among males [36–38]. Due to these differences, our analysis focused on males and female groups using different cut-offs (1.68 and 2.3 respectively) to categorize the data.

However, the study presents many weaknesses, first among them the fact that it is based only on self-reports so there are no external observations (e.g., clinician’s diagnosis or objective informant data, etc.) corroborating or refusing the data collected. The cross-sectional design of this study prevents causal interpretations regarding the relationship between body appreciation, social influences and EDs risks. Further studies, e.g. with longitudinal design, should better investigate whether body dissatisfaction precedes ED symptoms.

Some biases may arise from the use of EDE-Q as it has been highlighted that this tool may better assess ED specific symptomatology among females (i.e., thinness-oriented attitudes and behaviors) than symptomatology more common among males (i.e., muscularity-oriented attitudes and behaviors) [36, 110]. Future studies are needed to better understand the most common symptomatology in men, including additional tools assessing symptoms associated with muscularity concerns.

Another limitation of the study is that the risk factors we examined may be more indicative of the binge-eating and purging subtype of anorexia nervosa (AN), characterized by a dysregulated profile (such as smoking, alcohol consumption, and high BMI), or of bulimia and binge-eating disorder (BED), rather than of the restrictive subtype of AN. Given these considerations, we believe it is important to conduct further research that specifically investigates risk factors associated with individual eating disorders (such as bulimia, BED, etc.), and particularly with anorexia nervosa (AN) and ARFID. This is especially relevant considering the critical need for early diagnosis of eating disorders, which is essential for improving treatment outcomes.

## Conclusions

To our knowledge, this is the first study in Italy to collect data on EDs risk factors in a large sample of general school population, aged 14–16 year, using widely used self-report standardized questionnaires.

Eating disorders are confirmed as a health concern among Italian young people and may represent an underestimated issue in adolescents. Limited information is available to identify risk factors in adolescents for EDs, representing an obstacle for earlier identification of people at risk for EDs. As this study highlights, the EDE-Q, BAS and FBVS tests have shown to be valuable, practical and easily deployable self-administered screening tools to better understand risk factors and behaviors that contribute to make individuals more vulnerable to developing these psychiatric conditions. In order to better provide early identification and to improve surveillance of EDs, further studies with a larger sample size and standardized design are needed to assess the causal nature of the risk factors identified in the current research. Such studies might help implementing the development of targeted, evidence-based prevention, early intervention, and treatment programs.

## Electronic supplementary material

Below is the link to the electronic supplementary material.

Supplementary Material 1

## Data Availability

No datasets were generated or analysed during the current study.
