# Supplementary material for: Social and individual factors associated with eating disorders risk among adolescents in secondary schools of Sicily (south-Italy)
Source: Child Adolesc Psychiatry Ment Health. 2025 Jul 14;19:75. doi: 10.1186/s13034-025-00940-2 (PMC12257751; doi:10.1186/s13034-025-00940-2)
Supplement: Supplementary file 1 — Supplementary Material 1 [file 13034_2025_940_MOESM1_ESM.docx]

**Supplementary Materials**

- 1. *Sample recruitment*

During the school year 2022/23, 15 secondary second degree schools of Palermo and Catania were selected on the basis of their Principals’ availability. The total population of the schools was 3,150 students: 69 classes for a total of 1,740 students (55.2% of total involved school population) were randomly selected for the study to estimate the prevalence of EDs and some of their related risk factors. Inclusion criteria for study participation were: age comprised between 14 and 16 years old; attending the first, second or third class.

*1.2. Gender specific regression models*

Generalized linear regression analysis splitting the data sample by gender were conducted. Three different models were generated using EDE-Q score as outcome and BMI, BAS, presence of physical exercise, alcohol consumption, smoking habits, self-harm episodes, body comparison throughout social media, presence of victimization and bullyism, impact of COVID and presence of EDs in family as continuous predictors. The results are shown in the Table S1.

**Table S1.** Generalized linear models results splitting by gender using EDE-Q as dependent variable.

| GENDER | MODEL | UnStd. Coefficients | | Std. Coefficients | t | pvalue |
| --- | --- | --- | --- | --- | --- | --- |
|  |  | T | Std. Error | Beta |  |  |
| Non binary | (Constant) | -2.857 | 4.453 |  | -,642 | ,587 |
|  | BMI | ,138 | ,165 | ,484 | ,837 | ,491 |
|  | BAS | -1.106 | ,570 | -,894 | -1.942 | ,192 |
|  | Sport | 5.168 | 1.851 | 1.793 | 2.792 | ,108 |
|  | Acohol consumption | -,643 | 1.340 | -,226 | -,480 | ,679 |
|  | Smoking | -1.621 | 1.617 | -,562 | -1.002 | ,422 |
|  | Self-Harm | ,263 | 2.175 | ,091 | ,121 | ,915 |
|  | Social comparison | -,205 | 1.021 | -,072 | -,201 | ,860 |
|  | Victimization | 4.230 | 1.789 | 1.421 | 2.364 | ,142 |
|  | Bullying | ,561 | 1.343 | ,161 | ,418 | ,717 |
|  | COVID-19 impact | 1.291 | 2.253 | ,453 | ,573 | ,624 |
|  | Familiar EDs | -1.076 | ,950 | -,377 | -1.132 | ,375 |
| Females | (Constant) | 1.139 | ,238 |  | 4.780 | **,000** |
|  | BMI | ,061 | ,009 | ,166 | 6.640 | **,000** |
|  | BAS | -,646 | ,036 | -,505 | -17.752 | **,000** |
|  | Sport | ,139 | ,064 | ,051 | 2.183 | **,029** |
|  | Acohol consumption | ,257 | ,072 | ,094 | 3.583 | **,000** |
|  | Smoking | -,020 | ,075 | -,007 | -,273 | ,785 |
|  | Self-Harm | ,297 | ,083 | ,088 | 3.558 | **,000** |
|  | Social comparison | ,333 | ,071 | ,122 | 4.719 | **,000** |
|  | Victimization | ,068 | ,084 | ,020 | ,808 | ,419 |
|  | Bullying | -,077 | ,101 | -,018 | -,759 | ,448 |
|  | COVID-19 impact | ,510 | ,070 | ,187 | 7.334 | **,000** |
|  | Familiar EDs | ,133 | ,076 | ,043 | 1.761 | ,079 |
| Males | (Constant) | ,066 | ,182 |  | ,362 | ,717 |
|  | BMI | ,068 | ,007 | ,270 | 10.059 | **,000** |
|  | BAS | -,407 | ,027 | -,411 | -15.066 | **,000** |
|  | Sport | ,167 | ,053 | ,078 | 3.168 | **,002** |
|  | Acohol consumption | -,016 | ,049 | -,008 | -,322 | ,747 |
|  | Smoking | ,160 | ,061 | ,069 | 2.629 | **,009** |
|  | Self-Harm | ,293 | ,089 | ,083 | 3.298 | **,001** |
|  | Social comparison | ,173 | ,056 | ,076 | 3.073 | **,002** |
|  | Victimization | ,277 | ,078 | ,091 | 3.557 | **,000** |
|  | Bullying | ,132 | ,060 | ,056 | 2.208 | **,028** |
|  | COVID-19 impact | ,327 | ,052 | ,167 | 6.293 | **,000** |
|  | Familiar EDs | ,194 | ,054 | ,089 | 3.559 | **,000** |

BMI: Body Mass Index (Kg/cm^2^); BAS: Body Appreciation Scale; EDs: Eating Disorders; Std: standardized; UnStd: UnStandardized.

*1.3. Sensitivity analysis bullism/victimism*

Generalized linear regression analyses were conducted, stratifying individuals based on their involvment in bullying either as pepetrators or as victims. The global EDE-Q score was used as the outcome variable. Predictor variables included BMI, BAS score, engagement in physical excersise, alcohol consumption, smoking habits, episodes of self-harm, body comparison throughout social media, impact of COVID-19 and presence of EDs in family. The results are presented in the Figure S1.


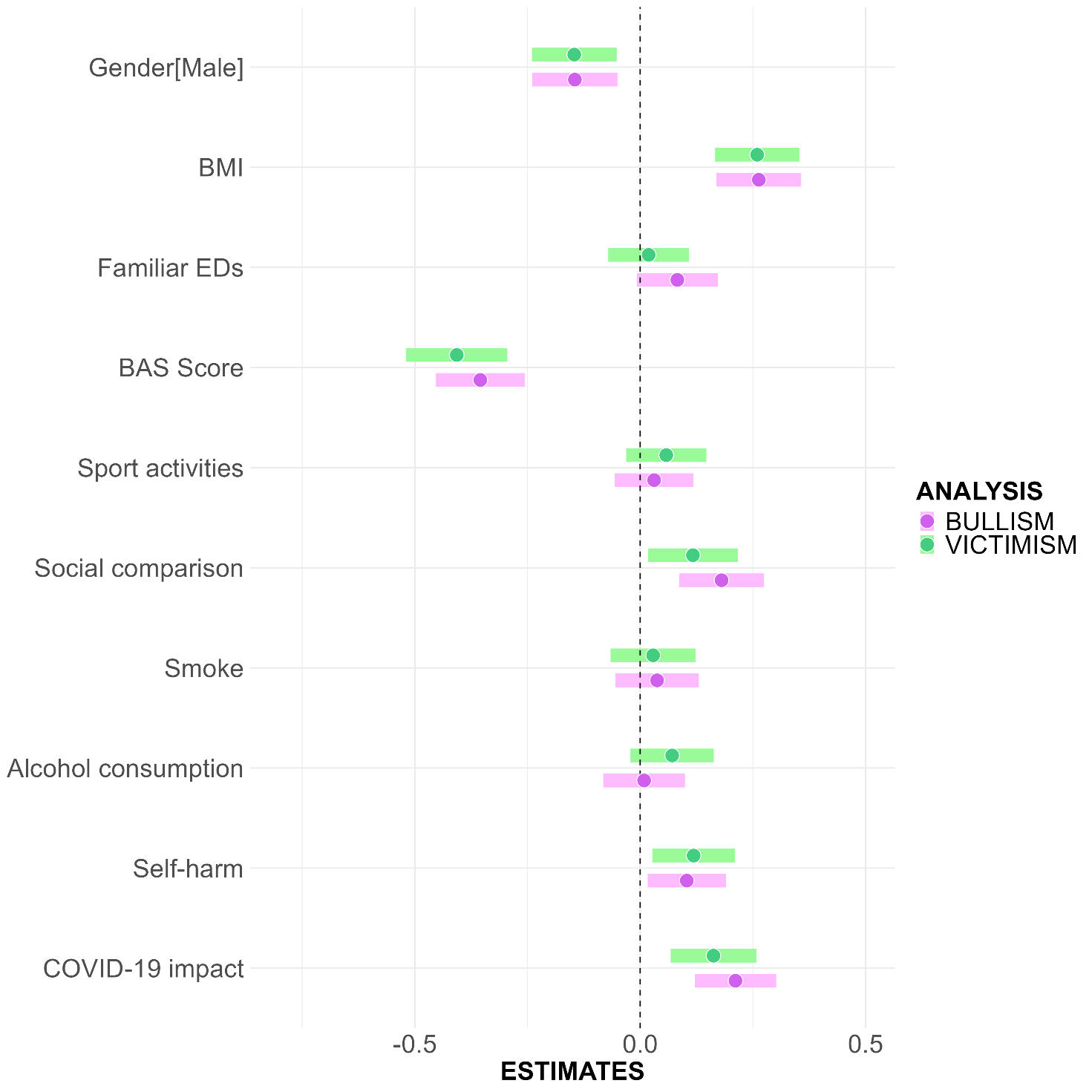


**Figure S1.** Standardized beta coefficients (with 95% confidence intervals) from generalized linear regression models assessing the association between individual and psychosocial factors and EDE-Q global score, stratified by involvement in bullying. Green bars represent individuals who reported being victims of bullying, and pink bars represent those who reported being perpetrators.
